# (*E*)-1-(2,4-Dinitro­phen­yl)-2-pentyl­idenehydrazine

**DOI:** 10.1107/S1600536810002102

**Published:** 2010-01-30

**Authors:** Patrícia D. Neunfeldt, Auri R. Duval, Wilson Cunico, Solange M. S. V. Wardell, Edward R. T. Tiekink, James L. Wardell

**Affiliations:** a Departamento de Química Orgânica, Universidade Federal de Pelotas (UFPel), Campus Universitário, s/n°, Caixa Postal 354, 96010-900 Pelotas, RS, Brazil; b CHEMSOL, 1 Harcourt Road, Aberdeen AB15 5NY, Scotland; c Department of Chemistry, University of Malaya, 50603 Kuala Lumpur, Malaysia; d Centro de Desenvolvimento Tecnológico em Saúde (CDTS), Fundação Oswaldo Cruz (FIOCRUZ), Casa Amarela, Campus de Manguinhos, Av. Brasil 4365, 21040-900 Rio de Janeiro, RJ, Brazil

## Abstract

The title compound, C_11_H_14_N_4_O_4_, is essentially planar with an r.m.s. deviation for the 19 non-H atoms of 0.152 Å. The conformation about the C=N bond is *E*, and the mol­ecule has a U-shape as the butyl group folds over towards the aromatic system. An intra­molecular C—H⋯N inter­action occurs. The crystal packing is dominated by N—H⋯O hydrogen bonding and C—H⋯O contacts, leading to twisted zigzag supra­molecular chains along the *c* direction. The crystal packing brings two nitro O atoms into an unusually close proximity of 2.686 (4) Å. While the nature of this inter­action is not obvious, there are several precendents for such short nitro–nitro O⋯O contacts of less than 2.70 Å in the crystallographic literature.

## Related literature

For background to the biological uses of hydrazones, see: Rollas & Küçükgüzel (2007 ▶). For background to the synthesis, see: Furniss *et al.* (1999 ▶); Neuenfeldt *et al.* (2009 ▶). For a description of the Cambridge Structural Database, see: Allen (2002 ▶).
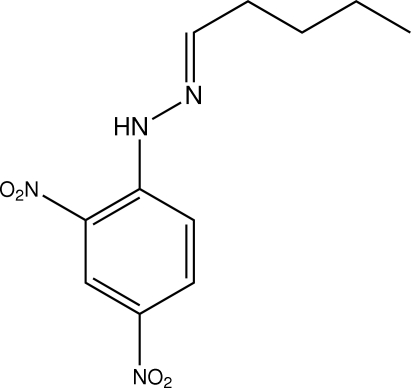


## Experimental

### 

#### Crystal data

C_11_H_14_N_4_O_4_
*M* *_r_* = 266.26
Monoclinic, 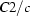

*a* = 31.162 (3) Å
*b* = 4.4930 (4) Å
*c* = 18.7329 (14) Å
β = 106.159 (4)°
*V* = 2519.2 (4) Å^3^
*Z* = 8
Mo *K*α radiation
μ = 0.11 mm^−1^
*T* = 120 K
0.32 × 0.03 × 0.02 mm

#### Data collection

-Nonius KappaCCD area-detector diffractometer
Absorption correction: multi-scan (*SADABS*; Sheldrick, 2007 ▶) *T*_min_ = 0.628, *T*_max_ = 1.000
8172 measured reflections
2174 independent reflections
1451 reflections with *I* > 2σ(*I*)
*R*_int_ = 0.115

#### Refinement

*R*[*F* ^2^ > 2σ(*F* ^2^)] = 0.078
*wR*(*F* ^2^) = 0.183
*S* = 1.10
2174 reflections
179 parameters
H atoms treated by a mixture of independent and constrained refinement
Δρ_max_ = 0.28 e Å^−3^
Δρ_min_ = −0.27 e Å^−3^

### 

Data collection: *COLLECT* (Hooft, 1998 ▶); cell refinement: *DENZO* (Otwinowski & Minor, 1997 ▶) and *COLLECT*; data reduction: *DENZO* and *COLLECT*; program(s) used to solve structure: *SHELXS97* (Sheldrick, 2008 ▶); program(s) used to refine structure: *SHELXL97* (Sheldrick, 2008 ▶); molecular graphics: *ORTEP-3* (Farrugia, 1997 ▶) and *DIAMOND* (Brandenburg, 2006 ▶); software used to prepare material for publication: *publCIF* (Westrip, 2010 ▶).

## Supplementary Material

Crystal structure: contains datablocks global, I. DOI: 10.1107/S1600536810002102/fj2271sup1.cif

Structure factors: contains datablocks I. DOI: 10.1107/S1600536810002102/fj2271Isup2.hkl

Additional supplementary materials:  crystallographic information; 3D view; checkCIF report

## Figures and Tables

**Table 1:** Hydrogen-bond geometry (Å, °)

*D*—H⋯*A*	*D*—H	H⋯*A*	*D*⋯*A*	*D*—H⋯*A*
N1—H1n⋯O4	0.87 (4)	1.99 (4)	2.616 (5)	128 (3)
N1—H1n⋯O4^i^	0.87 (4)	2.41 (4)	3.166 (5)	146 (4)
C3—H3⋯O1^ii^	0.95	2.39	3.335 (5)	176
C6—H6⋯N2	0.95	2.40	2.735 (5)	100

Symmetry codes: (i) 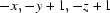; (ii) 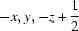.

**Fig. 1.**
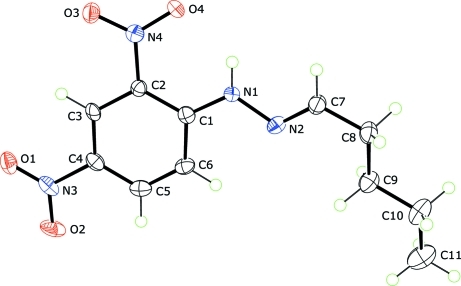
The molecular structure of (I) showing the atom-labelling scheme and displacement ellipsoids at the 50% probability level.

**Fig. 2.**
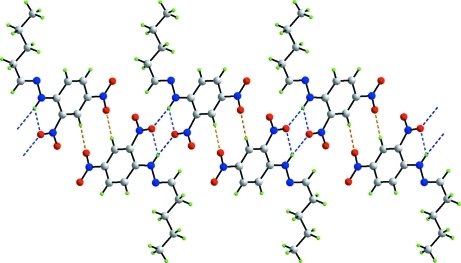
A view of the supramolecular chain in (I) mediated by N–H···O hydrogen bonding and C–H···O contacts, shown as blue and orange dashed lines, respectively. Colour code: O, red; N, blue; C, grey; and H, green.

**Fig. 3.**
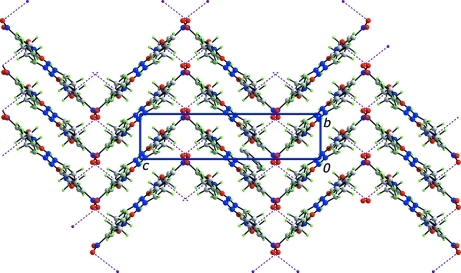
A view of the supramolecular 2-D array in (I) with N–O···π interactions shown as purple dashed lines. This figure highlights the zigzag topology of the chains shown in Fig. 2. Colour code: O, red; N, blue; C, grey; and H, green.

**Fig. 4.**
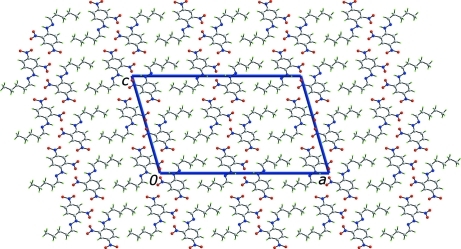
A view of the stacking of layers (illustrated in Fig. 3) in (I) with the interdigitation of the n-butyl residues. Colour code: O, red; N, blue; C, grey; and H, green.

## supplementary crystallographic information

### Comment

2,4-Dinitrophenylhydrazine is a frequently used reagent for the characterization of aldehydes and ketones (Furniss *et al.*, 1999). The 2,4-dinitrophenylhydrazone products are generally formed readily in good yield and purity. The ready formation of 2,4-dinitrophenyl hydrazones of carbonyl compounds can be a disadvantage as found during the attempted formation of a thiazolidinone from 2,4-dinitrophenylhydrazine, pentanal and mercaptoacetic acid, using a similar one-pot synthesis to that used successfully with amines, carbonyl compounds and mercaptoacetic acid (Neuenfeldt *et al.*, 2009). Instead of the targeted thiazolidinone derivative, the 2,4-dinitrophenylhydrazone of pentanal was isolated in very high yield: as shown below, this compound was efficiently produced from a reaction mixture reaction just involving 2,4-dinitrophenylhydrazine and pentanal. Hydrazones containing the –NHN=CH moiety constitute an important class of antimicrobial, anticonvulsant, analgesic, antiinflammatory, antiplatelet, antitubercular and antitumoral agents. (Rollas & Küçükgüzel, 2007).

To a first approximation, the molecule of (I), Fig. 1, is flat with the maximum deviations of torsion angles from the ideal 0 or 180 ° being 9.0 (7) ° for N2–C7–C8–C9, and -170.7 (4) ° for C1–N1–N2–C7; the r.m.s. deviation of the non-hydrogen atoms = 0.152 Å. The *n*-butyl side-chain folds over to be oriented towards the benzene ring. The conformation about the C7═N3 bond [1.270 (5) Å] is *E*. In the crystal packing, supramolecular chains are formed along the *c* direction. These are sustained by four-membered {···H···O}_2_ synthons as the amine-H1n atom is bifurcated forming intra- and intermolecular *N*–*H*···Onitro hydrogen bonds, Fig. 2 and Table 1. Additional stabilization to the chain is afforded by ten-membered {···ONC_2_H}_2_ synthons, Fig. 2 and Table 1. Whereas the smaller of the synthons is disposed about a centre of inversion, the larger has crystallographic 2-fold symmetry and has a distinct folded conformation. The latter induces considerable kinks in the chain as emphasized in Fig. 3 which illustrates the formation of 2-D arrays *via* N–O···π interactions [N–O3···*Cg*(C1–C6)^i^ = 3.163 (3) Å with an angle at O3 = 89.9 (2) ° where *Cg* is the ring centroid of the C1–C6 ring and symmetry operation *i* = *x*, -1 + *y*, *z*]. Globally, the layers formed in the *bc* plane stack along the *a* direction with interdigitation of the saturated residues. It is noted that the packing of molecules brings into close proximity two nitro-O atoms, *i.e.* O4···O4^ii^ = 2.686 (4) Å for *ii*: -*x*, 1 - *y*, 1 - *z*. While the nature of this interaction is not obvious, there are approximately 50 precendents for such O_nitro_···O_nitro_ contacts < 2.70 Å in the crystallographic literature (Allen, 2002).

### Experimental

A mixture of 2,4-dinitrophenylhydrazine 1 (3 mmol) and pentanal 2 (3 mmol) in toluene (35 ml) was heated at 403 K with a Dean-Stark trap for 3 h. The reaction was cooled and the crude product was recrystallized from ethanol, yield 69%. m.p. 371–372 K. ^1^H NMR (400 MHz, CDCl_3_): d 11.00 (br, 1H, NH), 9.11 (d, 1H, J = 2.4 Hz), 8.29 (dd, 1H, J = 9.6 and 2.4 Hz), 7.93 (d, 1H, J = 9.6 Hz), 7.54 (t, 1H, J = 5.2 Hz), 2.43 (m, 2H), 1.60 (m, 2H), 1.43 (sext, 2H, J = 7.6 Hz), 0.97 (t, 3H, J = 7.6 Hz) p.p.m.

### Refinement

The C-bound H atoms were geometrically placed (C–H = 0.95–0.99 Å) and refined as riding with *U*_iso_(H) = 1.2–1.5*U*_eq_(C). The methyl H atoms were rotated to fit the electron density. The N–H atom was located in a difference map and refined with *U*_iso_(H) = 1.2*U*_eq_(N). The reported structure, while unambiguous, is not optimal owing to the poor quality of the crystals available for analysis.

### Crystal data

**Table:**

C_11_H_14_N_4_O_4_	*F*(000) = 1120
*M**_r_* = 266.26	*D*_x_ = 1.404 Mg m^−^^3^
Monoclinic, *C*2/*c*	Mo *K*α radiation, λ = 0.71073 Å
Hall symbol: -C 2yc	Cell parameters from 14843 reflections
*a* = 31.162 (3) Å	θ = 2.9–27.5°
*b* = 4.4930 (4) Å	µ = 0.11 mm^−^^1^
*c* = 18.7329 (14) Å	*T* = 120 K
β = 106.159 (4)°	Needle, yellow
*V* = 2519.2 (4) Å^3^	0.32 × 0.03 × 0.02 mm
*Z* = 8	

### Data collection

**Table:**

-Nonius KappaCCD area-detector diffractometer	2174 independent reflections
Radiation source: Enraf Nonius FR591 rotating anode	1451 reflections with *I* > 2σ(*I*)
10 cm confocal mirrors	*R*_int_ = 0.115
Detector resolution: 9.091 pixels mm^-1^	θ_max_ = 25.0°, θ_min_ = 3.0°
φ and ω scans	*h* = −36→36
Absorption correction: multi-scan (*SADABS*; Sheldrick, 2007)	*k* = −5→5
*T*_min_ = 0.628, *T*_max_ = 1.000	*l* = −22→22
8172 measured reflections	

### Refinement

**Table:**

Refinement on *F*^2^	Primary atom site location: structure-invariant direct methods
Least-squares matrix: full	Secondary atom site location: difference Fourier map
*R*[*F*^2^ > 2σ(*F*^2^)] = 0.078	Hydrogen site location: inferred from neighbouring sites
*wR*(*F*^2^) = 0.183	H atoms treated by a mixture of independent and constrained refinement
*S* = 1.10	*w* = 1/[σ^2^(*F*_o_^2^) + (0.0406*P*)^2^ + 14.6755*P*] where *P* = (*F*_o_^2^ + 2*F*_c_^2^)/3
2174 reflections	(Δ/σ)_max_ < 0.001
179 parameters	Δρ_max_ = 0.28 e Å^−^^3^
0 restraints	Δρ_min_ = −0.27 e Å^−^^3^

### Special details

**Table:**

Geometry. All s.u.'s (except the s.u. in the dihedral angle between two l.s. planes) are estimated using the full covariance matrix. The cell s.u.'s are taken into account individually in the estimation of s.u.'s in distances, angles and torsion angles; correlations between s.u.'s in cell parameters are only used when they are defined by crystal symmetry. An approximate (isotropic) treatment of cell s.u.'s is used for estimating s.u.'s involving l.s. planes.
Refinement. Refinement of *F*^2^ against ALL reflections. The weighted *R*-factor *wR* and goodness of fit *S* are based on *F*^2^, conventional *R*-factors *R* are based on *F*, with *F* set to zero for negative *F*^2^. The threshold expression of *F*^2^ > 2σ(*F*^2^) is used only for calculating *R*-factors(gt) *etc*. and is not relevant to the choice of reflections for refinement. *R*-factors based on *F*^2^ are statistically about twice as large as those based on *F*, and *R*- factors based on ALL data will be even larger.

### Fractional atomic coordinates and isotropic or equivalent isotropic displacement parameters (Å^2^)

**Table:**

	*x*	*y*	*z*	*U*_iso_*/*U*_eq_	
O1	0.04985 (11)	−0.5722 (7)	0.23571 (16)	0.0294 (8)	
O2	0.11770 (10)	−0.4264 (8)	0.24360 (16)	0.0360 (9)	
O3	−0.03340 (10)	−0.1033 (7)	0.37999 (16)	0.0263 (8)	
O4	−0.00644 (9)	0.2652 (7)	0.45386 (15)	0.0240 (7)	
N1	0.07910 (11)	0.3841 (8)	0.49031 (18)	0.0194 (8)	
H1N	0.0551 (14)	0.401 (10)	0.505 (2)	0.023*	
N2	0.11897 (11)	0.5276 (8)	0.52633 (18)	0.0224 (9)	
N3	0.08254 (13)	−0.4163 (9)	0.26201 (19)	0.0267 (9)	
N4	−0.00244 (11)	0.0661 (8)	0.41033 (19)	0.0213 (8)	
C1	0.07833 (13)	0.2003 (10)	0.4333 (2)	0.0197 (10)	
C2	0.04022 (13)	0.0353 (10)	0.3940 (2)	0.0185 (9)	
C3	0.04158 (14)	−0.1701 (9)	0.3391 (2)	0.0191 (9)	
H3	0.0161	−0.2868	0.3159	0.023*	
C4	0.08074 (14)	−0.2003 (10)	0.3192 (2)	0.0222 (10)	
C5	0.11852 (14)	−0.0324 (10)	0.3534 (2)	0.0242 (10)	
H5	0.1450	−0.0528	0.3381	0.029*	
C6	0.11736 (14)	0.1613 (10)	0.4090 (2)	0.0230 (10)	
H6	0.1434	0.2733	0.4321	0.028*	
C7	0.11826 (14)	0.6629 (11)	0.5855 (2)	0.0248 (10)	
H7	0.0922 (15)	0.660 (11)	0.604 (2)	0.030*	
C8	0.15800 (14)	0.8254 (11)	0.6307 (2)	0.0281 (11)	
H8A	0.1693	0.7207	0.6788	0.034*	
H8B	0.1485	1.0266	0.6415	0.034*	
C9	0.19629 (14)	0.8581 (12)	0.5957 (2)	0.0292 (11)	
H9A	0.2041	0.6593	0.5802	0.035*	
H9B	0.1863	0.9830	0.5505	0.035*	
C10	0.23763 (15)	0.9966 (13)	0.6476 (3)	0.0382 (13)	
H10A	0.2293	1.1896	0.6655	0.046*	
H10B	0.2485	0.8654	0.6914	0.046*	
C11	0.27528 (16)	1.0479 (14)	0.6126 (3)	0.0481 (15)	
H11A	0.2826	0.8602	0.5921	0.072*	
H11B	0.3016	1.1216	0.6503	0.072*	
H11C	0.2660	1.1951	0.5727	0.072*	

### Atomic displacement parameters (Å^2^)

**Table:**

	*U*^11^	*U*^22^	*U*^33^	*U*^12^	*U*^13^	*U*^23^
O1	0.0374 (18)	0.0244 (18)	0.0233 (16)	0.0013 (17)	0.0035 (14)	−0.0058 (15)
O2	0.0333 (18)	0.050 (2)	0.0308 (17)	0.0130 (18)	0.0186 (15)	−0.0036 (17)
O3	0.0273 (16)	0.0281 (19)	0.0246 (15)	−0.0065 (15)	0.0090 (13)	−0.0059 (15)
O4	0.0244 (16)	0.0223 (18)	0.0265 (15)	0.0017 (13)	0.0093 (13)	−0.0074 (15)
N1	0.0163 (18)	0.019 (2)	0.0236 (18)	0.0000 (16)	0.0061 (15)	−0.0035 (17)
N2	0.0181 (17)	0.026 (2)	0.0231 (19)	−0.0029 (16)	0.0052 (15)	0.0018 (17)
N3	0.037 (2)	0.023 (2)	0.0218 (19)	0.010 (2)	0.0108 (18)	0.0006 (17)
N4	0.0228 (19)	0.020 (2)	0.0219 (18)	0.0005 (17)	0.0078 (16)	0.0023 (18)
C1	0.022 (2)	0.017 (2)	0.019 (2)	0.0026 (19)	0.0042 (18)	0.0026 (19)
C2	0.019 (2)	0.017 (2)	0.019 (2)	−0.0002 (18)	0.0047 (18)	0.0006 (19)
C3	0.024 (2)	0.010 (2)	0.021 (2)	0.0042 (18)	0.0025 (18)	0.0015 (18)
C4	0.029 (2)	0.018 (2)	0.021 (2)	0.005 (2)	0.0091 (19)	0.0028 (19)
C5	0.022 (2)	0.025 (3)	0.028 (2)	0.005 (2)	0.0105 (19)	0.009 (2)
C6	0.019 (2)	0.022 (3)	0.028 (2)	−0.0008 (19)	0.0082 (19)	0.006 (2)
C7	0.023 (2)	0.027 (3)	0.024 (2)	−0.004 (2)	0.0054 (19)	−0.003 (2)
C8	0.030 (2)	0.028 (3)	0.027 (2)	−0.008 (2)	0.009 (2)	−0.005 (2)
C9	0.027 (2)	0.033 (3)	0.027 (2)	−0.006 (2)	0.006 (2)	0.001 (2)
C10	0.032 (3)	0.043 (4)	0.033 (3)	−0.010 (2)	−0.002 (2)	0.004 (3)
C11	0.032 (3)	0.056 (4)	0.054 (3)	−0.008 (3)	0.009 (3)	0.010 (3)

### Geometric parameters (Å, °)

**Table:**

O1—N3	1.221 (5)	C5—H5	0.9500
O2—N3	1.238 (4)	C6—H6	0.9500
O3—N4	1.236 (4)	C7—C8	1.483 (6)
O4—N4	1.240 (4)	C7—H7	0.96 (4)
N1—C1	1.345 (5)	C8—C9	1.521 (6)
N1—N2	1.396 (5)	C8—H8A	0.9900
N1—H1N	0.87 (4)	C8—H8B	0.9900
N2—C7	1.270 (5)	C9—C10	1.515 (6)
N3—C4	1.458 (5)	C9—H9A	0.9900
N4—C2	1.451 (5)	C9—H9B	0.9900
C1—C2	1.420 (6)	C10—C11	1.513 (6)
C1—C6	1.423 (5)	C10—H10A	0.9900
C2—C3	1.391 (6)	C10—H10B	0.9900
C3—C4	1.378 (5)	C11—H11A	0.9800
C3—H3	0.9500	C11—H11B	0.9800
C4—C5	1.395 (6)	C11—H11C	0.9800
C5—C6	1.365 (6)		
			
C1—N1—N2	118.9 (3)	N2—C7—C8	121.3 (4)
C1—N1—H1N	119 (3)	N2—C7—H7	122 (3)
N2—N1—H1N	122 (3)	C8—C7—H7	117 (3)
C7—N2—N1	114.4 (3)	C7—C8—C9	115.5 (4)
O1—N3—O2	124.8 (4)	C7—C8—H8A	108.4
O1—N3—C4	118.7 (3)	C9—C8—H8A	108.4
O2—N3—C4	116.6 (4)	C7—C8—H8B	108.4
O3—N4—O4	122.6 (3)	C9—C8—H8B	108.4
O3—N4—C2	119.2 (3)	H8A—C8—H8B	107.5
O4—N4—C2	118.2 (3)	C10—C9—C8	113.0 (4)
N1—C1—C2	124.0 (4)	C10—C9—H9A	109.0
N1—C1—C6	120.1 (4)	C8—C9—H9A	109.0
C2—C1—C6	115.8 (4)	C10—C9—H9B	109.0
C3—C2—C1	122.5 (4)	C8—C9—H9B	109.0
C3—C2—N4	115.9 (4)	H9A—C9—H9B	107.8
C1—C2—N4	121.6 (4)	C11—C10—C9	114.0 (4)
C4—C3—C2	118.5 (4)	C11—C10—H10A	108.7
C4—C3—H3	120.8	C9—C10—H10A	108.7
C2—C3—H3	120.8	C11—C10—H10B	108.7
C3—C4—C5	121.3 (4)	C9—C10—H10B	108.7
C3—C4—N3	118.8 (4)	H10A—C10—H10B	107.6
C5—C4—N3	119.8 (4)	C10—C11—H11A	109.5
C6—C5—C4	119.8 (4)	C10—C11—H11B	109.5
C6—C5—H5	120.1	H11A—C11—H11B	109.5
C4—C5—H5	120.1	C10—C11—H11C	109.5
C5—C6—C1	121.9 (4)	H11A—C11—H11C	109.5
C5—C6—H6	119.0	H11B—C11—H11C	109.5
C1—C6—H6	119.0		

### Hydrogen-bond geometry (Å, °)

**Table:**

*D*—H···*A*	*D*—H	H···*A*	*D*···*A*	*D*—H···*A*
N1—H1n···O4	0.87 (4)	1.99 (4)	2.616 (5)	128 (3)
N1—H1n···O4^i^	0.87 (4)	2.41 (4)	3.166 (5)	146 (4)
C3—H3···O1^ii^	0.95	2.39	3.335 (5)	176
C6—H6···N2	0.95	2.40	2.735 (5)	100

Symmetry codes:  (i) −*x*, −*y*+1, −*z*+1; (ii) −*x*, *y*, −*z*+1/2.
